# Sonification of Computer Processes: The Cases of Computer Shutdown and Idle Mode

**DOI:** 10.3389/fnins.2022.862663

**Published:** 2022-05-04

**Authors:** Claudio Panariello, Roberto Bresin

**Affiliations:** Sound and Music Computing, KTH Royal Institute of Technology, Stockholm, Sweden

**Keywords:** sonification, software processes, aesthetic, glitch, Paetzold recorder

## Abstract

Software is intangible, invisible, and at the same time pervasive in everyday devices, activities, and services accompanying our life. Therefore, citizens hardly realize its complexity, power, and impact in many aspects of their daily life. In this study, we report on one experiment that aims at letting citizens make sense of software presence and activity in their everyday lives, through sound: the invisible complexity of the processes involved in the shutdown of a personal computer. We used sonification to map information embedded in software events into the sound domain. The software events involved in a shutdown have names related to the physical world and its actions: write events (information is saved into digital memories), kill events (running processes are terminated), and exit events (running programs are exited). The research study presented in this article has a “double character.” It is an artistic realization that develops specific aesthetic choices, and it has also pedagogical purposes informing the causal listener about the complexity of software behavior. Two different sound design strategies have been applied: one strategy is influenced by the sonic characteristics of the Glitch music scene, which makes deliberate use of glitch-based sound materials, distortions, aliasing, quantization noise, and all the “failures” of digital technologies; and a second strategy based on the sound samples of a subcontrabass Paetzold recorder, an unusual and special acoustic instrument which unique sound has been investigated in the contemporary art music scene. Analysis of quantitative ratings and qualitative comments of 37 participants revealed that the sound design strategies succeeded in communicating the nature of the computer processes. Participants also showed in general an appreciation of the aesthetics of the peculiar sound models used in this study.

## 1. Sonification of Computer Processes

Software is intangible, invisible, and at the same time present in all electronic devices, activities, and services which are part of our everyday life. This makes it hard for a layperson to realize its complexity, power, and impact in many aspects of their daily life. Sound is the perfect medium for representing the invisible. For example, it is successfully used in movies and theater to communicate to the audience events that are happening outside the visual field, such as footstep sounds of people approaching the scene, or opening and closing doors.

In this article, we report on a study that aims at letting laypersons make sense of software presence and activity in their everyday lives, through sound: the invisible complexity of the software activities involved in the idle mode of a personal computer and its shutdown. In previous study, researchers have used different sonification strategies, e.g., for real-time monitoring of web server activities (Barra et al., 2002), of server logfiles (Hauer and Vogt, 2015), for communicating the network's traffic and monitoring its security (Vickers et al., 2017b; Debashi and Vickers, 2018; Axon et al., 2019), for monitoring the software trace collected when performing a copy and paste command in a text editor (Thomas et al., 2018). Different aesthetics have been used in these sonification studies, such as MIDI synthesizers, granular synthesis, and samples of a human voice whispering the sentence “copy and paste” manipulated using the Max/MSP's SPAT Operator.

As a matter of fact, the relation between sonification and its aesthetic is non trivial: “The fact that a sonification can be heard musically, then, risks our attention being directed away from its data-informational function and toward features such as the sonic textures, the beauty of the sound and so forth.” (Vickers et al., 2017a). Therefore, the sonification can have an aesthetic connotation, as long as this one does not interfere with the communication of the data relations, i.e., not interfere with the objectivity of the sonification. In fact, as pointed out in Vickers (2016), “if the goal of communicating data gets subverted in sonification projects, [...], composers engaging in sonification music will often lose sight of that goal altogether in pursuit of aesthetic interest.”

In the present study, we used sonification to map the information embedded in software processes into the sound domain as described in the following sections, developing sound models that deliberately take into account aesthetic reflections, while keeping clear in mind the aim to objectively communicate data relationships. The software processes that we considered for the present study are the computer *idle mode* and the computer *shutdown*. These are processes common to all electronic devices and, therefore, have general applicability. A device is in *idle mode* when the user has not interacted with it for a certain time interval and it is waiting for a user- or software-generated event to start a new process and to exit the idle mode. The *shutdown* process is a process that shuts down an electronic device so that it is no longer active and it needs to be restarted in order to work. This is not to be confused with the case when a user forces a device to switch-off by pressing the on-off button for a few seconds. Besides being two of the processes common to all devices, their meaning and properties can also be associated with other domains. In fact, a shutdown process involves names that are related to the physical world and its actions: write events (information is saved into digital memories), kill events (running processes are terminated), and exit events (running programs are exited). Therefore, the research study presented in this article has a “double character”: it is an artistic realization that develops specific aesthetic choices, and it has also pedagogical purposes informing the causal listener about the complexity of software behavior.

## 2. Sound Design

The sonification of a personal computer shutdown starts from a very large amount of data: a collection of all the processes going on when a computer receives the instruction to shutdown. Since the time distance between two collected events was 10^−8^s in general, the data were grouped into larger windows of time (0.1*s*) to make it more manageable from an audio point of view. The sound design process was organized in different cycles. In the beginning, a mere translation of the events into the audio domain was done by means of sine waves with a percussive envelope. This was a fairly simple sonification which aim was to familiarize with the data and to look for possible musical patterns for further investigation.

Two different aesthetics were then adopted to realize the final sonifications: the first one made use of only synthesized sounds taking inspiration from Glitch music (refer to section 2.1); the second one made use of sound samples of a peculiar acoustic instrument, the subcontrabass Paetzold recorder (refer to section 2.2).

### 2.1. Sonification With Synthesized Material: Glitches

**Figure 1:**
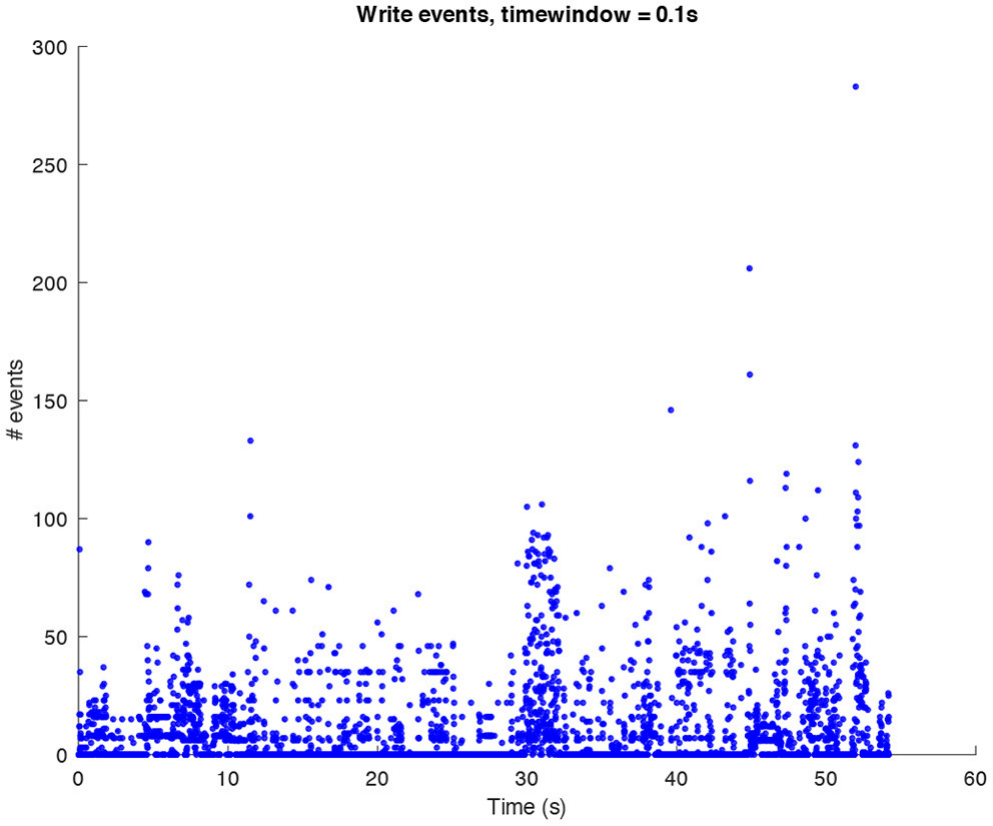
Amount of *write* events per window of time (0.1*s*) in a shutdown process.

**Figure 2:**
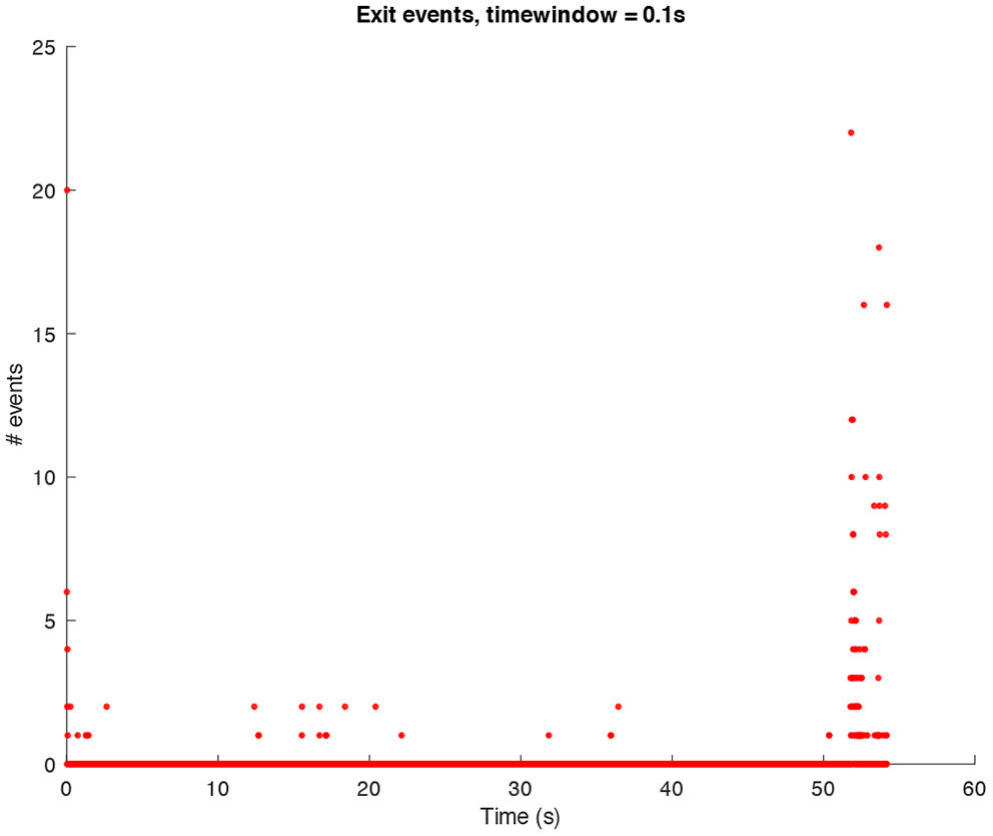
Amount of *exit* events per window of time (0.1*s*) in a shutdown process.

**Figure 3:**
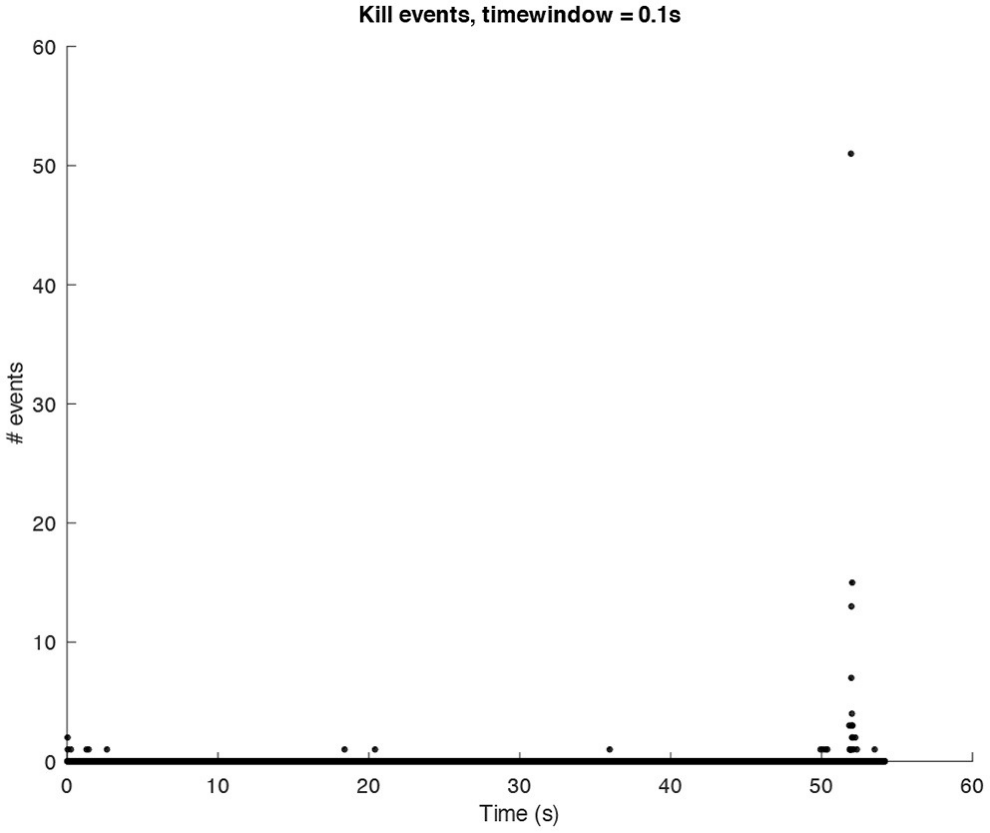
Amount of *kill* events per window of time (0.1*s*) in a shutdown process.

In the following sound design stages, ideas about how we would have liked the final result to sound started to be part of the process. This does not mean that the data were changed in order to achieve a particular sound output, rather it affected the design of the synthetic sounds then used for the sonification. We decided to look at the sonic characteristics of the Glitch (and post-Glitch) scene. Conceptually, Glitch music is a genre of electronic music that started emerging in the early 1990s making deliberate use of glitch-based sound materials, distortions, aliasing, quantization noise, and all the “failures” of digital technologies (Cascone, 2000). Thus, it seemed interesting to try and use this aesthetic to sonify the computer shutdown using a dramaturgy of the “computer death.” This approach was also inspired by the data itself since they contain all the processes recorded when a shutdown is launched and categorized as “write,” “exit,” and “kill” events (refer to Figures 1–3 for three plots of these events).

In the latest stage of the sound design, the one presented here, the sonification is a result of four interconnected sound layers. The first three ones are used for the communication of *write* processes, *exit*, and *kill* events collected in the shutdown, and the fourth one is a sound communicating the density of the three events altogether over time. The sonification was realized in SuperCollider^1^.

The sonification of the *write* events makes use of a granulated harmonic sum of sine waves and of the UGen Gendy1, a dynamic stochastic generator conceived by Xenakis (1992) and coded in SuperCollider by Collins (2011). The fundamental frequency of the sine waves the size of the grains, and the overall sound level are all mapped (in different ways) to the number of *write* events per window of time. *Write* events are seen here as positive events, since they are saving data on the server for future use, and we, therefore, sonify them using high frequency sounds which are characteristic of the communication of positive emotions such as happiness (Bresin and Friberg, 2011). The result is a high pitched glitch texture characterized by an almost constant level of activity for the entire shutdown process, due to the continuous writing processes, and with an increase of activity toward the end, when the majority of write processes accumulate.

The sonification of *kill* events is created by means of a sine wave with a parametric envelope with a sharp attack and variable release time. Since the distribution of the *kill* events is quite sparse, we have decided to map the number of these events per window of time (0.1s) not only to sound level and fundamental frequency but also to the actual number of sounds overlapping. In this case, the fundamental frequency has been chosen in the low range (centered around 40*Hz*). This choice was based on results from previous studies on expressive communication in music performance which have shown how sadness and fear can be characterized by low frequencies (Bresin and Friberg, 2011). The result is an erratic and menacing layer of low hits, interesting from a mere musical point of view since it involuntarily increases the overall drama.

The sonification of *exit* events is created with a bandpass filter applied to pink noise multiplied by an impulse with decay. The aim was to create sharp explosive glitch sounds. Also here, the number of *exit* events per window of time has been mapped to the filter's central frequency and to impulse frequency. The frequency used in this case was in the middle-high range (500−1, 000*Hz*). Since *exit* and *kill* events are connected, the flow of exit glitches follows the low layer of the *kill* ones, creating a sort of musical counterpoint.

Finally, the fourth layer is a drone created by a filtered granulation of an input sound made up of 6 summed harmonic sine waves. The total number of events over time has been used as a control signal for the fundamental sine waves' frequency, the filter cutoff frequency, and the size and amplitudes of the grains, following indications from a previous overview study on sonification in which it was found that size variations can be sonified by using timbral changes (Dubus and Bresin, 2013).

The final sonification, i.e., the superposition of the four layers, has been played reading the data at about 3 times faster than the original speed since the sparsity of *kill* and *exit* events would have led to a great amount of silence in the sonification. The soundfile is available as Supplementary Sound 1.

**Figure 4:**
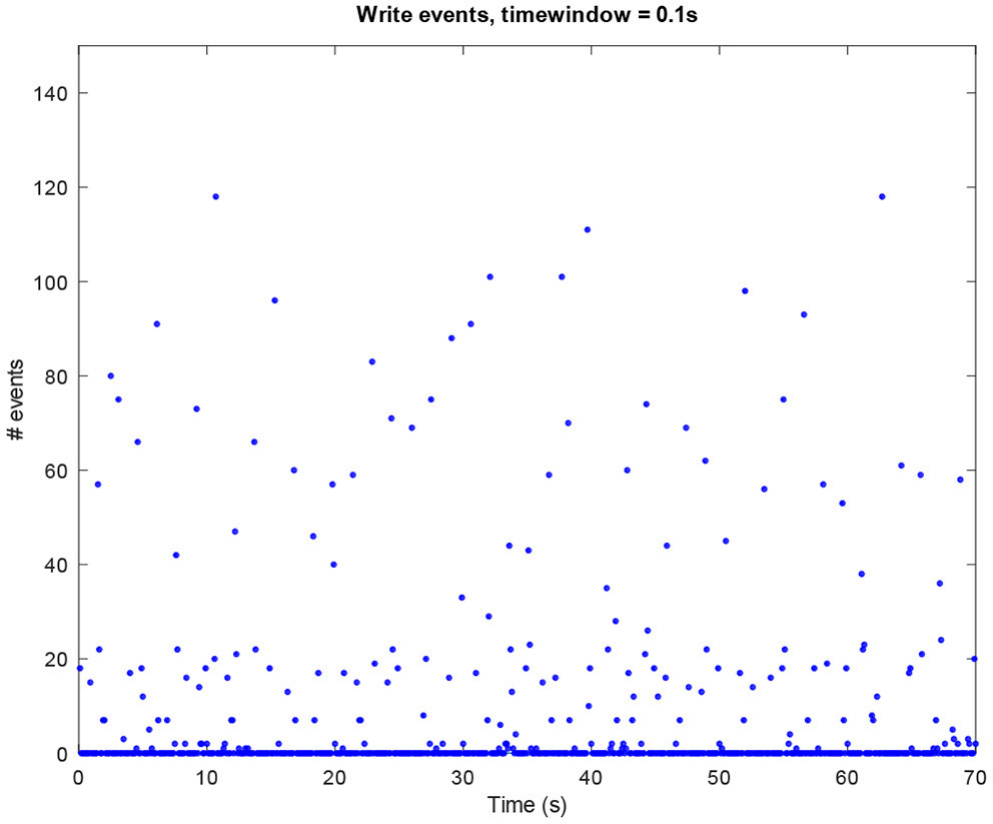
Amount of *write* events per window of time (0.1*s*) in an idle mode process.

**Figure 5:**
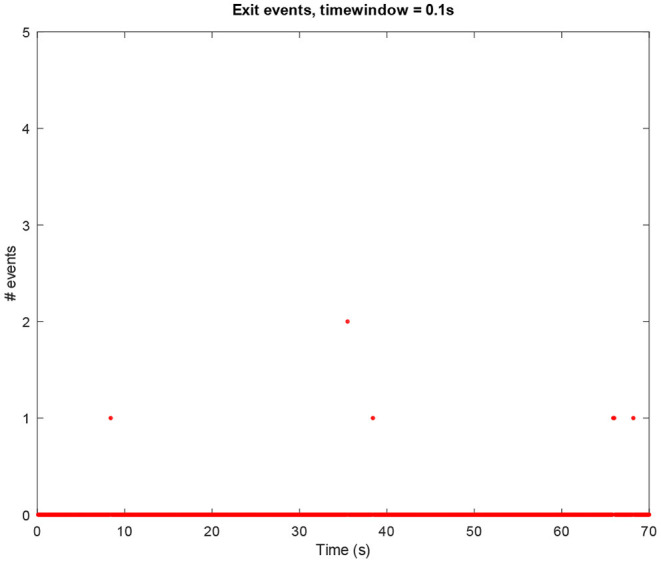
Amount of *exit* events per window of time (0.1*s*) in an idle mode process.

**Figure 6:**
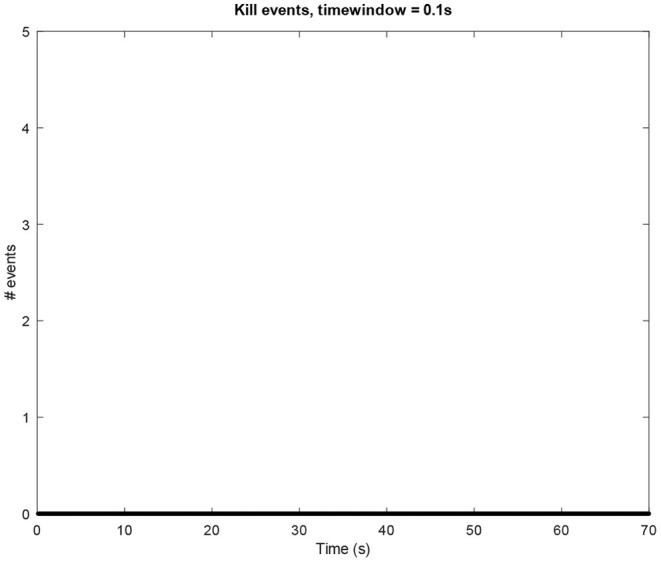
Amount of *kill* events per window of time (0.1*s*) in an idle mode process.

The above-presented sonification strategy for *write, kill*, and *exit* events have been kept unaltered when using idle mode computer data. Figures 4–6 show the plots for these three kinds of events in an idle mode process lasting 70 s. It can be immediately noticed how the overall computer activity is very different when compared to the plots of shutdown data presented in Figures 1–3: Figure 4 shows few and almost regularly distributed *write* events, with an average of 7.65 events per window of time of 0.1*s*, which is ten times less than the 75.57 events per window of time shown in Figure 1; Figure 5 shows extremely sparse *exit* events, occurring only at 8, 35, and 65 s; and finally Figure 6 shows no *kill* events at all. As in the case of shutdown data sonification, also here, a fourth layer containing the sum of all the events per window of time has been used. Since the scarcity of *exit* events and the absence of *kill* events, this layer basically contains *write* events. The sonification of these idle mode data results in a calm, quiet, and almost periodical soundscape where the high pitched texture created by the *write* events is matched by the drone-like low frequency fourth layer, with very few occasional sound pops given by the *exit* events. The sonification of computer idle mode data is available as Supplementary Sound 2.

As seen, the main purpose was to use a one-to-many strategy, in which the same data are controlling more structures. In the reading of the data, it has been chosen to have windows of time large enough to understand aurally what was going on, as magnifying a process.

### 2.2. Sonification With Sampled Material: Paetzold Recorder

The Paetzold recorder is a square-pipe recorder that bears its name from Joachim Paetzold, a German designer and manufacturer, who built the first models in 1953^2^. Since 2012, the Paetzold recorders are known as “Paetzold by Kunah” and they are produced in tenor, bass, greatbass, contrabass, subgreatbass, and subcontrabass sizes.

For this sonification, the sounds of a subcontrabass Paetzold recorder were used, recorded by Antonio Politano at HEMU. The sounds are available online and freely downloadable^3^.

There are several reasons why we decided to use such a special and unusual instrument. We wanted to explore a sample-based sonification to have a sort of non-synthesized counterpart to the glitch sonification, still with very well-defined sonic characteristics. We also wanted to use the timbral potentialities of such an instrument and the iconic underground dark world aesthetic that it brings with itself and that has truly fascinated late twentieth and twenty-first-century contemporary composers, who have dug into this instrument's sound world, especially in combination with the use of electronics [refer to for example studies by Romitelli (Arbo, 2014), Vaggione, Di Scipio (Di Scipio, 2005)]. As a matter of fact, as it can be read on the website of PRIME-Paetzold Recorder Investigation for Music and Electronics from the HEMU: “*The Paetzold square recorder was developed to offer amateurs and professionals a cheap instrument with a sound more stable than one of the traditional recorders. In fact, not only the Paetzold square recorder meets these requirements, but it has an extremely rich palette of sounds, which makes it a very interesting instrument for contemporary music”* with its “*large palette of noises, whistles and rustles, the delicacy of its tonal and dynamical nuances, its multiphonics, glissandi and flageolets.”*

In this second sonification, the sound samples have been used in a way that they could resemble plausible gestures on the subcontrabass Paetzold recorder (e.g., the labium opening, which is a very idiomatic gesture on this instrument), thus not denaturing the instrumental characteristics and creating a final sonification that could echo a plausible piece for Paetzold recorder(s).

As for the glitch sonification, also the sonification with the Paetzold is the result of four interconnected sound layers (*write, exit*, and *kill* events, plus the sum of all events over a window of time). This sonification was realized in SuperCollider as well.

The sonification of *write* events makes use of 15 different samples of staccato overblow on the fundamentals^4^ F_1_ (≈ 44 Hz) and A_1_ (≈ 55 Hz) ordered by increasing dynamic, thus resulting in sounds with increasing harmonic content. The samples and their repetitions have been mapped to the number of *write* events per window of time: in this way, the higher the number, the higher the spectral content of the sound will be and the faster the sample will be repeated. The samples' amplitude has also been linearly mapped to the number of *write* events. An additional low-pass filter on the samples, in which cutoff frequency is also mapped to the number of *write* events, creates more spectral variety. The overall result of the sonification of the *write* events is a sequence of almost continuous but rhythmically variable repeated staccato overblows that resemble the similar peculiar idiomatic gesture on a Paetzold recorder.

The sonification of *kill* events makes use of three samples of “overblow transitions,” i.e., three different overblow sharp attacks followed by a short decay of the fundamental. These samples, their playback rate, and their amplitudes have all been linearly mapped to the number of *kill* events per window of time.

The sonification of *exit* events makes use of a sample of “reverse slap,” which is a sound produced by a tongue action similar to the slap one but performed while breathing in rather than breathing out. The result is a different percussive sound compared to the traditional slap, smoother in timbre and with a slightly longer attack. The reverse slap playback rate and amplitude have been linearly mapped to the number of *exit* events per window of time.

As in the case of the sonification with glitches, we have here as well a fourth layer created using a sample of a long sustained low note (F_1_), filtered by a lowpass filter. The sample's amplitude and the filter cutoff frequency are controlled by the overall number of events over time.

The final sonification, as before, is the superposition of the four layers. Also here, it has been played reading the data at about 3 times faster than the original speed. The soundfile is available as Supplementary Sound 3.

The sonification of 70 s of computer idle mode data using these second aesthetics is available as Supplementary Sound 4.

### 2.3. Incoherent Mapping

Besides the sonification of shutdown and idle mode data with two different aesthetics as described in sections 2.1, 2.2, we decided to create other sonifications based on perceptually incoherent mappings, that is we swapped the mappings of *write, kill* and *exit* events used in the sonification presented in the previous sections. More in detail, in these new sonifications, we decided to use the sound model developed for the *write* events to sonify *exit* events; to use the sound model developed for *exit* events to sonify the total sum of events per window of time; to use the sound model developed for *kill* events to sonify *write* events; and finally to use the sound model developed for the total sum to sonify *kill* events. These four incoherent mapping sonifications are available online and in the Supplementary Material ^5^.

Such incoherent mappings are expected to sound less appropriate than the initial intended mappings since the data are now mapped to sounds using an arbitrary mapping not meant to be the “correct” one.

## 3. Experiment

We run a perceptual experiment^6^ with the main aim of evaluating and comparing both the functionality and the aesthetical qualities communicated by the different sonifications.

### 3.1. Participants

The survey was shared with students and colleagues at KTH Royal Institute of Technology, on mailing lists, and social networks. Participants were allowed to re-distribute the survey to anyone they believed would be interested in participating in the study.

### 3.2. Stimuli

Stimuli were soundfiles created from the sonification of shutdown data and idle mode data, using the two different aesthetics and *incoherent mapping*, thus having four stimuli for the shutdown and four stimuli for the idle mode. The total of eight stimuli is described in detail as follows:

- Stimulus 1: sonification of computer shut-down with glitch aesthetics as described in section 2.1;
- Stimulus 2: same as stimulus 1 but with *incoherent mapping* as described in section 2.3;
- Stimulus 3: sonification of computer shut-down with a second aesthetics as described in section 2.2;
- Stimulus 4: same as stimulus 3 but with *incoherent mapping*;
- Stimulus 5: sonification of computer idle mode with glitch aesthetics as described in section 2.1;
- Stimulus 6: same as stimulus 5 but with *incoherent mapping*;
- Stimulus 7: sonification of computer idle mode with a second aesthetics as described in section 2.2;
- Stimulus 8: same as stimulus 7 but with *incoherent mapping*.

The stimuli were cut from the full shutdown and idle mode sonifications and they were lasting about 20 s each.

### 3.3. Procedure

Participants were asked to listen to sounds created from data of a computer shutdown (i.e., when a computer closes all software programs in preparation to turn off its power) vs. data of a computer idle mode. Before starting the listening task, they were given a definition of what shutdown and idle mode mean.

The survey was then divided into two parts. In the first part, each page presented two sounds from the shutdown and from the idle mode categories. Participants were asked to listen to them and to select the sound they thought best represented the sonification of shutdown computer data. The task was repeated 16 times with all the available stimuli. The presentation order of the stimuli was randomized for each participant. In the second part of the survey, participants were instead asked to rate the pleasantness of the sonifications, divided into shutdown sonifications and idle mode ones. They could rate each of the sonification on a scale from “Not pleasant at all” (0) to “Very pleasant” (100) with a step size of 1. Besides this, participants were also asked to leave a comment on the sounds they just heard.

## 4. Results

A total of 37 persons (14 F, 22 M, 1 prefer not to answer, average age 36.84 years) completed the experiment. Regarding self-reported musical expertise, 14 described themselves as “experts or full-professional activity,” 8 as “semi-professional activity (several years of practice, skills confirmed),” 5 as “some experience (advanced amateur, some years of practice),” 4 as “little experience (occasional amateur),” and 6 as having “no experience.”

Pooling data^7^ across sound model and stimuli resulted in a total overall accuracy of 55.07%. A one-sample χ^2^ test suggested that overall accuracy was not significantly different from chance. Total accuracy per coherent mapping when pooling data across stimuli was 60.14%. A one-sample χ^2^ test suggested that accuracy was above chance [χ^2^_(1, *n* = 296)_ = 12.16, *p* < 0.001]. Total accuracy per sound model when pooling data across stimuli was 61.49% for the coherent mapping glitch sound model, 58.78% for the coherent mapping Paetzold recorder sound model, 47.97% for the incoherent mapping glitch sound model, and 52.02% for the incoherent mapping Paetzold recorder sound model.

In order to evaluate if musical experts vs. non-experts differed in accuracy, we assigned data for persons identifying as musical experts or semi-professionals to one category, and persons not identifying as musical experts or semi-professional to another one. This resulted in one group of 22 experts and 15 non-experts. Pooling data across sound models and stimuli pairs, overall accuracy was 57.67% for experts and 51.25% for non-experts. A χ^2^ test revealed no significant difference in accuracy between the two groups. Considering only the coherent mappings, accuracy was 65.91.% for experts and 51.66% for non-experts. A χ^2^ test revealed a significant relationship between the two variables. Experts are more likely than non-experts to spot the shutdown [χ2_(1, *N* = 296)_ = 6.037, *p* < 0.05].

**Figure 7:**
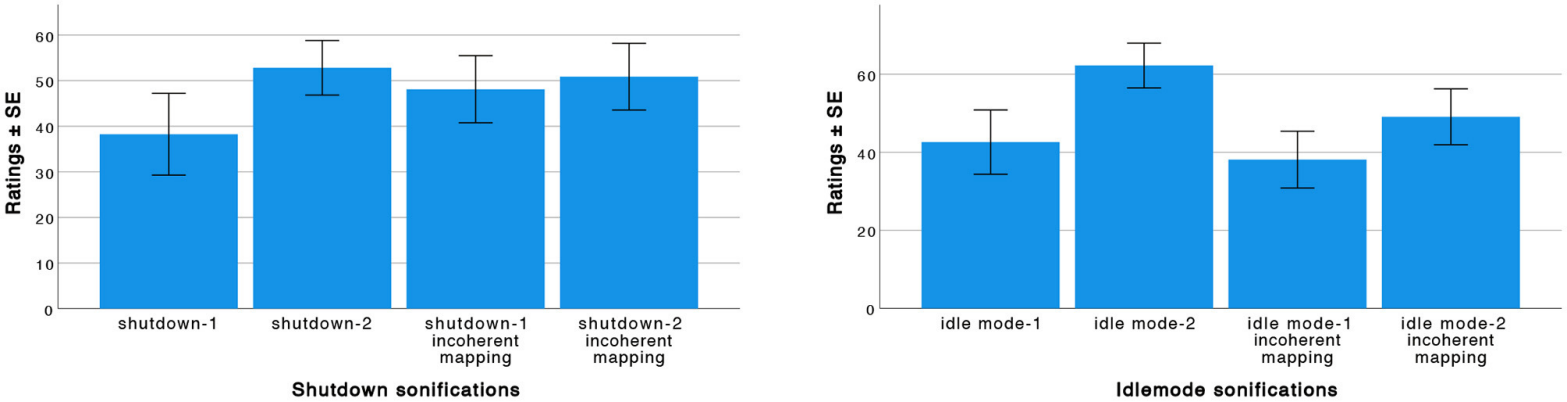
Pleasantness: mean ratings and SE for the eight sonifications. “1” stands for the first aesthetic (glitch sound model) and “2” stands for the second aesthetic (Paetzold recorder sound model).

We conducted a one-way repeated measures ANOVA on the data from the second part of the experiment to compare the pleasantness ratings of the four shutdowns and the four idle mode sonifications (refer to Figure 7). Mauchly's test on the shutdown sonifications ratings indicated that the assumption of sphericity had been violated, $\chi_{\left(5\right)}^{2}=17.460$, *p* = 0.004, therefore, degrees of freedom were corrected using Huynh-Feldt estimates of sphericity (ϵ = 0.844). The results show that there is a significant effect of the sound model on the pleasantness rating, *F*_(2.53, 91.10)_ = 3.34, *p* < 0.05. Pairwise comparisons based on estimated marginal means (with Bonferroni corrections) indicated that the shutdown sonification with coherent mapping realized with the second aesthetic (Paetzold recorder sound model) was rated (*M* = 52.81, *SD* = 18.18) significantly higher than the coherent mapping realized with the first aesthetic (glitch sound model) (*M* = 38.24, *SD* = 27.29). On the other side, it was not rated significantly different from both the incoherent mapping sonifications.

Mauchly's test on the idle mode sonifications ratings indicated that the assumption of sphericity had not been violated, $\chi_{\left(5\right)}^{2}=8.307$, *p* = 0.140. The results show that there is a significant effect of the sound model on the pleasantness rating, *F*_(3, 108)_ = 12.52, *p* < 0.05. Pairwise comparisons based on estimated marginal means (with Bonferroni corrections) indicated that the idle mode sonification with coherent mapping realized with the second aesthetic (Paetzold recorder), was rated (*M* = 62.22, *SD* = 17.43) significantly different from the other three sonifications.

The open text question provided some insights into the quality of sounds. Overall, despite commenting on the subjectivity of the pleasantness, participants agreed on the aesthetic quality of the sonifications, defining them as “*great,” “interesting from a musical/sound design perspective,” “inspired by M. Subotnick,”* or just “*these sound effects are sick”* [sic]. At least two participants described the shutdown sonifications as “*amazing”* but “*unsettling”* or “*disturbing”* at the same time. Regarding the idle mode sonifications, participants wrote that “*the mellow, lower sounds are more reassuring”* [sic], “*the calm temporal regularity is good”*, and that “*the static of this computer event allows to appreciate the nature of the sounds themselves”*. A participant also commented on the fact that in his opinion an idle sound should “be of an ‘ambient' nature and not draw attention” [sic], and added that two of the presented idle mode sonifications were going in that aesthetic direction. However, the majority of participants agreed in finding the high-pitched sounds as “*not pleasant”*, “*disturbing in different ways”*, defining them as “*insect-like timbre”* that “*contributed to my lower rating”*, and that they would have preferred “*softer timbres”*. On the other side, participants agreed on the pleasantness of the low frequency sounds.

Some participants commented on the difficulty to associate such sonifications to computer processes, and in particular to the shutdown one: one participant wrote that “*If they are supposed to be communicating any kind of information it is lost on me”*, and another one that “*It was difficult to judge which sound corresponds to a computer shutting down”*. Moreover, a third participant commented that “*shutting down is quite rapid, and these sounds are rather long”*, while a fourth one that “*I expect the Shutdown sound of a computer to be quick and not intrusive”*.

Few participants mentioned that they were able to detect rhythm and/or periodic figures in the shutdown sonifications and that this aspect made them like or not the sounds; in addition, two participants wrote that they were able to spot a “*contrabass clarinet”* and a “*Shakuhachi flute sound”* in the shutdown sonifications.

## 5. Discussion

The current study aimed at exploring the use of particular sound aesthetics in the sonification of computer processes (namely shutdown processes and idle mode processes). In terms of recognizing the shutdown sonification independently from the mappings used, we found that the overall accuracy was not significantly different from chance, while it became significant when considering only the coherent mappings. This can be explained considering that in the overall accuracy, the incoherent mappings are counted, and, as explained in Section 2.3, incoherent mappings are expected to sound less appropriate, i.e., we expected them to be less easily recognized since they were based on “wrong” mappings. Considering the accuracy of the coherent mappings of the expert vs. non-expert group, findings suggest that the former are more likely than the latter in recognizing the shutdown. This can be explained by taking into consideration the two particular aesthetics used for the sonifications: using two sound models characterized by strong sound qualities and almost niche aesthetic connotations (the glitches on one side, and the unusual acoustic instrument on the other) could have disoriented the non-expert group in the listening task.

**Figure 8:**
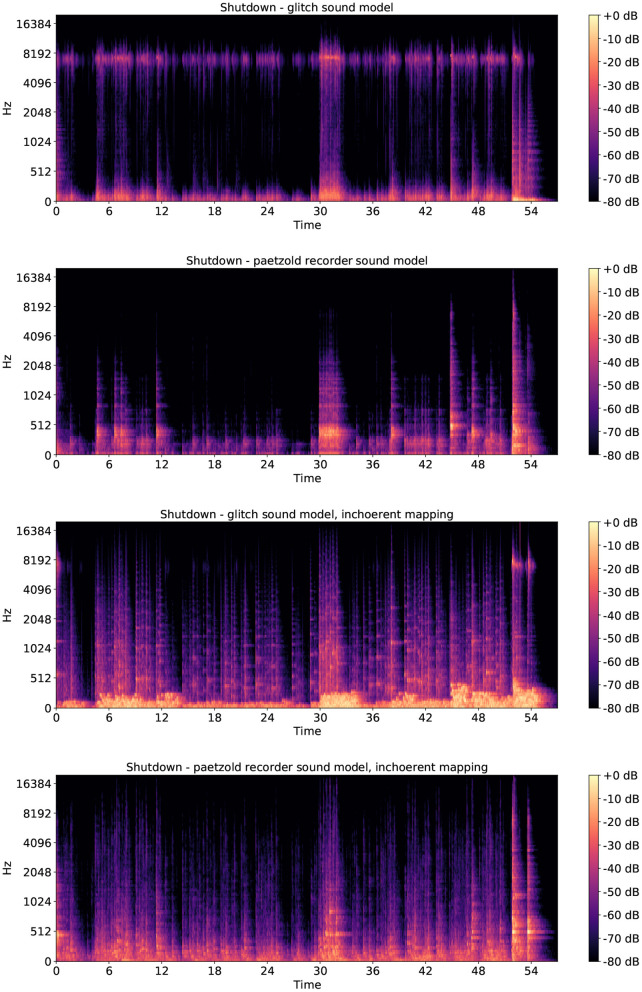
Spectrograms of the four computer shutdown sonifications. From top to bottom: shutdown glitch sound model, shutdown Paetzold recorder sound model, shutdown glitch sound model incoherent mapping, shutdown Paetzold recorder sound model incoherent mapping.

**Figure 9:**
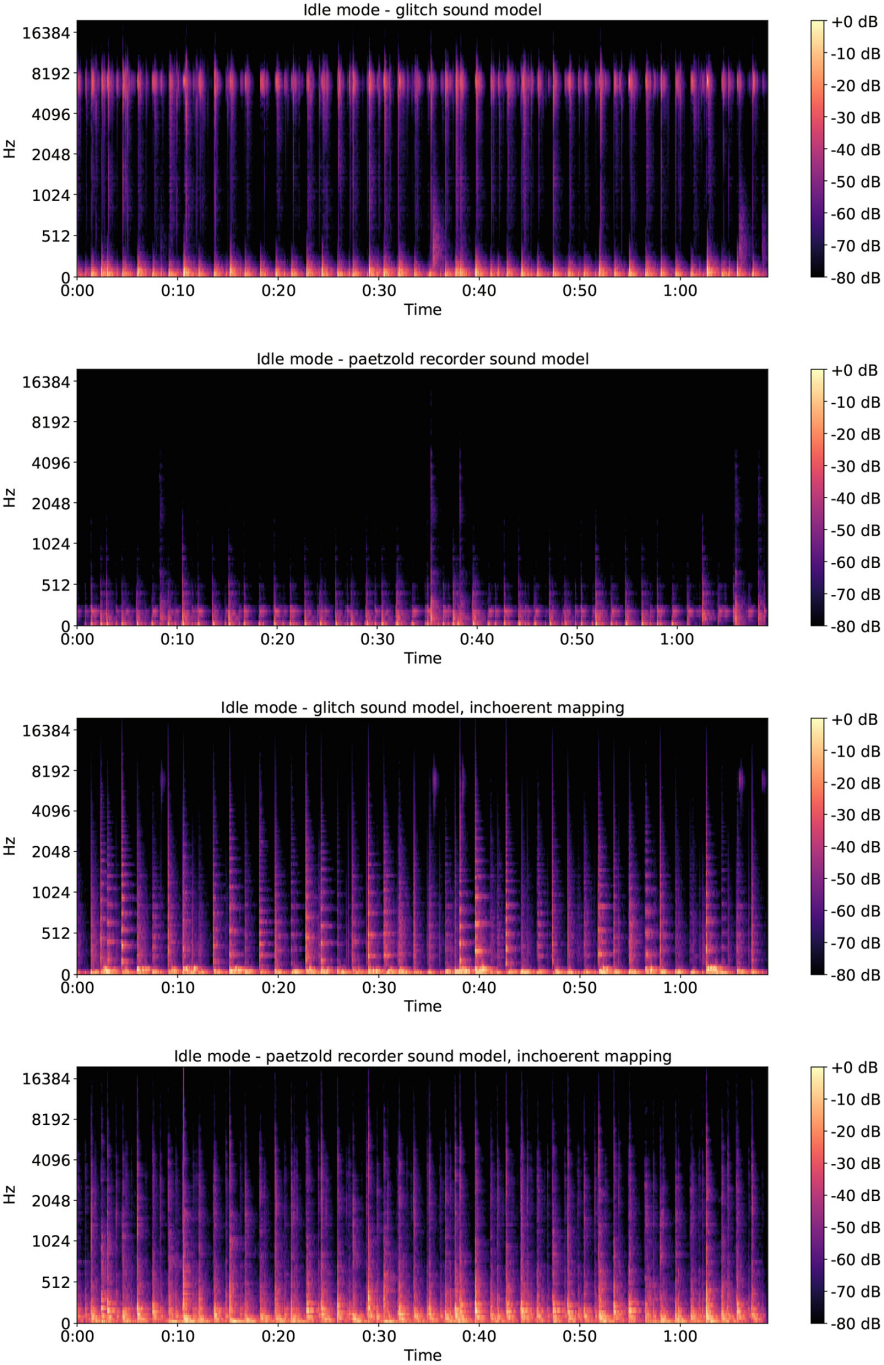
Spectrograms of the four computer idle mode sonifications. From top to bottom: idle mode glitch sound model, idle mode Paetzold recorder sound model, idle mode glitch sound model incoherent mapping, idle mode Paetzold recorder sound model incoherent mapping.

Based on the quantitative ratings, we can conclude that in the case of the shutdown ratings the Paetzold recorder sound model was preferred to the glitch sound model. Interestingly, it was not preferred to both of the incoherent mappings. This can be explained by reading the comments provided in the open-text box: the majority of the participants complained about the high-pitched sounds, finding them unpleasant. As a matter of fact, looking at the spectrograms of four shutdown sonifications shown in Figure 8, it can be noticed how the high and almost constant texture in the glitch sonification disappears in the two incoherent sonifications, due to the different mapping. This change in the spectral sound quality might have influenced the pleasantness ratings in favor of the incoherent mappings. Moving to the case of the idle mode ratings, quantitative findings suggest that the Paetzold recorder sound model was most preferred. Reading the comments provided in the open-text box, reasons to explain the preference for this sound model could be traced to its softer timbres compared to the glitch one, and in the preponderance of low frequency sounds compared to the incoherent mapping versions, as it can be noticed looking at the idle mode spectrograms shown in Figure 9.

Qualitative findings suggest that overall, the participants appreciated the sonifications, positively commenting on their sound qualities and musicality, and also in some cases, recognizing the sources of inspiration (both in terms of aesthetic and sound material). This appreciation motivates us to keep exploring complex and peculiar sound aesthetics in future sonifications. Moreover, the qualitative findings show the same trend as the quantitative ones, i.e., a preference for the Paetzold recorder sound model. This tendency can be explained not only referring to the presence of the high-pitched texture in the glitch sound model, but also to the fact that the glitch sounds are all synthetic, while the other model is based on samples performed on an acoustic musical instrument: the recognition of the presence of human gestures in the quality of the sounds could have influenced the rating (Leman, 2007). Besides that, it should also be considered that the glitch sound model was based on an aesthetic that uses the “failures” of digital technologies to create a music discourse based on noises and distortions: this aspect might have fooled the participants with less music expertise, prompting them to think that the glitch-based sonifications contained errors and they were “wrong.”

Concerning the incoherent mapping sonifications, the shuffling we made in the association between data events and sounds inevitably messed up with the expressive associations described in section 2, e.g., the one between *kill* events and low frequencies, thus also altering the overall expressive communication of the sonifications. This could explain the lower recognition rate of the shutdown sonifications based on incoherent mappings.

Comments on the difficulty to associate the sonifications to computer processes can be explained by the unfamiliarity of the internal computer activity, especially in the case of the shutdown. This finding is interesting because it motivates us to conduct more work in this direction, unveiling the hidden complexity of computers and software through the medium of sound.

Furthermore, in informal listening sessions with documentary director Erik Gandini^8^, for whom we did the sonification of a computer shutdown, and with other practitioners in the film making field, the coherent sonifications of the shutdown were interpreted as the sonic communication of a “sudden death.”

## 6. Conclusion

In this article, we presented exploratory work on the sonification of common software processes, shutdown and idle mode, by means of two aesthetically complex sound models. One model was inspired by the Glitch music scene and another by the subcontrabass Paetzold recorder. The main aim was to design aesthetically interesting sonifications which could raise awareness of the complexity of inner and hidden computer activity. Results from a listening experiment suggest that the sound models presented in this study succeeded in communicating the nature of the two software processes. Participants also showed in general an appreciation of the aesthetics of the peculiar sound models used in this study.

These promising results suggest future investigations involving the sonification of other computer processes and software, and its possible use in the creation of aesthetically complex soundscapes informing users about hidden activities of digital devices in general.

Additionally, both results and the methods developed in this project will be of importance for the design of future applications based on sonification in many areas, making use of a large amount of data where this discipline is gaining popularity, such as HCI, gaming, design, data journalism, rehabilitation, sports, robotics, electrical vehicles, automation, and education. In all these fields, the sonic display of information has the potential of adding a further dimension, augmenting the feedback usually provided by, e.g., visual display alone. For example, a well-designed sonic display can help make sense in a direct and intuitive way of the complexity and very large dimensions of information otherwise inaccessible and hard to grasp by individuals.

## Data Availability Statement

The original contributions presented in the study are included in the article/Supplementary Material, further inquiries can be directed to the corresponding author/s.

## Ethics Statement

Ethical review and approval was not required for the study on human participants in accordance with the local legislation and institutional requirements. The patients/participants provided their written informed consent to participate in this study.

## Author Contributions

CP and RB contributed to conception and design of the study, performed the statistical analysis, and wrote sections of the manuscript. CP realized the sonifications and wrote the first draft of the manuscript. All authors contributed to manuscript revision, read, and approved the submitted version.

## Funding

The research presented in this paper has been funded by the NAVET center at the KTH Royal Institute of Technology, by a grant to Roberto Bresin by the Swedish Research Council (grant 2017-03979) and by NordForsk Nordic University Hub Nordic Sound and Music Computing Network-NordicSMC (project number 86892).

## Conflict of Interest

The authors declare that the research was conducted in the absence of any commercial or financial relationships that could be construed as a potential conflict of interest.

## Publisher's Note

All claims expressed in this article are solely those of the authors and do not necessarily represent those of their affiliated organizations, or those of the publisher, the editors and the reviewers. Any product that may be evaluated in this article, or claim that may be made by its manufacturer, is not guaranteed or endorsed by the publisher.

## Footnotes

^1^ https://supercollider.github.io/

^2^ http://www.primeresearch.ch/index.php?option=com_content&view=featured&Itemid=101&lang=en

^3^ http://www.primeresearch.ch/index.php?option=com_content&view=article&id=59&Itemid=172&lang=en

^4^Considering A_4_ as the central A of the piano at 440 Hz.

^5^Incoherent shutdown sonifications with glitches and with Paetzold recorder are respectively Supplementary Sounds 5, 6. Incoherent idle mode sonifications with glitches and with Paetzold recorder are, respectively, Supplementary Sounds 7, 8.

^6^The entire survey can be accessed at https://survey.alchemer.com/s3/6639763/Sonification-computer-events.

^7^The table with the data is available in the Supplementary Material.

^8^ https://www.imdb.com/name/nm0304171/
